# ClaMS: A Classifier for Metagenomic Sequences

**DOI:** 10.4056/sigs.2075298

**Published:** 2011-11-28

**Authors:** Amrita Pati, Lenwood S. Heath, Nikos C. Kyrpides, Natalia Ivanova

**Affiliations:** 1 Genome Biology Program, DOE Joint Genome Institute, Walnut Creek, CA 94598,; 2 Department of Computer Science, Virginia Tech, Blacksburg, VA 24061

## Abstract

ClaMS – “Classifier for Metagenomic Sequences” – is a Java application for binning assembled contigs in metagenomes using user-specified training sets and initial parameters. Since ClaMS trains on sequence composition-based genomic signatures, it is much faster than binning tools that rely on alignments to homologs; ClaMS can bin ~20,000 sequences in 3 minutes on a laptop with a 2.4 GH× Intel Core 2 Duo processor and 2 GB RAM. ClaMS is meant to be a desktop application for biologists and can be run on any machine under any Operating System on which the Java Runtime Environment can be installed.

## Introduction

Metagenome binning is the process of assigning nucleotide sequences in a metagenome to known taxonomic groups. Mapping sequences to their taxonomic groups of origin leads to better characterization of a metagenome, which facilitates the accomplishment of objectives such as genome assembly from metagenomes and assembly and annotation improvement. Existing binning methods can be characterized in two ways -- (1) Composition-based binning tools and homology-based binning tools (2) *ab initio* unsupervised classifiers and supervised/training-based classifiers. In unsupervised binning, a dataset is classified to pre-existing bins trained on genomic sequences without any interference or supervision from the user. In supervised binning, the user integrates additional known facts about the dataset into the binning process by participating in the training process – by specifying sequences for each training bin and/or selecting the taxonomic units to which the dataset must be binned. Homology-based classifiers such as MEGAN [1] rely on alignments of sequences to homologs and are extremely computation-intensive. For large metagenomic datasets sequenced using next-generation sequencing technologies, homology-based binning can be prohibitive in terms of time and computation. While existing composition-based binning tools (Phylopythia [2], TETRA [3]) are much faster than homology-based binning tools, they are mostly unsupervised, and their accuracy is limited since the information about the presence and abundance of specific phylogenetic populations is not used in the binning process, even though such information obtained by 16S rDNA amplicon analysis results is available for many metagenomic datasets. Even in the absence of rRNA amplicon analysis experiments, some intelligence about the constituent organisms of a metagenome can be obtained by a few iterations of *ab initio* binning. The objective of ClaMS is to integrate this information into the binning process thus achieving higher accuracy of binning, and to produce a desktop/laptop application that is platform-independent, fast, and easily usable by biologists.

## Principles

ClaMS works by characterizing a sequence with a signature vector that is derived from its composition and described as a de Bruijn chain (DBC) signature [4]. A double stranded DNA sequence is treated as a walk in a de Bruijn graph and artifacts such as the stationary distribution of the underlying Markov chain and the strength of connectivity of various graph-components to the graph are used to compute the DBC signature. The transition probability matrix of the underlying Markov chain of even a relatively short sequence can accurately predict its stationary distribution, and this property is exploited in the computation of DBC signatures. The DBC signature is highly conserved within a species while varying between species and this can be proved both mathematically and experimentally [4]. This property also manifests at higher taxonomic levels. It is more complex than the oligonucleotide frequency signatures used by Phylopythia and TETRA, and different from the interpolated Markov Models used by Phymm [5]. Since a DBC signature of order k incorporates information about k-mers and (k+1)-mers in its computation, it is much faster to train. While the greater amount of information used by applications such as Phylopythia and Phymm does mean higher accuracy, ClaMS is targeted for use on assembled contigs with supervision from the user and in this scenario, accuracy is not compromised. Pre-computed signatures at various word lengths (2-4) are included with ClaMS for all finished genomes. These signatures have been computed using the taxonomy and isolate genome sequences in IMG [6] and will be updated with each release of ClaMS or on request. The users can define training sequence sets either by clicking a node in the phylogenetic tree in the ClaMS-GUI or by uploading their own fasta files of sequences. For each sequence to be binned, its signature, which is a vector, is computed. This signature is compared individually with the centroid signatures of all training sets and the best match is declared as the bin for that sequence.

## Results and Discussion

To demonstrate the accuracy of binning using ClaMS, we binned a real metagenome and a simulated metagenome using ClaMS. The real metagenome, the Phrap-assembled phosphorus removal sludge metagenome (SLU) sampled from laboratory-scale bioreactor (IMG/M, taxon OID: 2000000000 [6]), is 56.6M bases long, has 60.45% GC, and contains 31,742 assembled contigs. The simulated metagenome, the assembled medium complexity simulated simMC dataset from FAMeS [7], has 15109 non-chimeric contigs that were 1000 bases or longer and candidates for binning using ClaMS. We evaluated the results using cross-validation of the binned contigs. In the case of simMC, the correct bins of the contigs were already known for cross-validation, in the case of SLU, best hits from Blast alignment were used to cross-validate bins.

**Figure 1:**
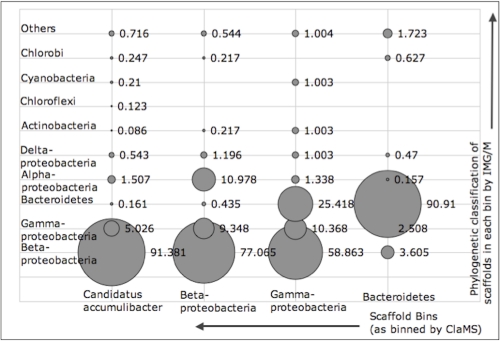
Cross-validation of scaffolds in the SLU dataset assigned by ClaMS to user-defined bins with respect to existing phylogenetic assignments of these scaffolds made by IMG/M based on their best Blast hit. The x-axis indicates the bin whose scaffolds are being cross-validated. The y-axis indicates the bacterial classes to which scaffolds in that bin actually map to in IMG/M. Bubble sizes represent percentages.

The phylogenetic distribution of genes in the SLU dataset based on their best Blast hits in IMG/M [6] and the 16S rRNA tree in [8] showed that the dataset was dominated by *Betaproteobacteria* (127 species), *Gammaproteobacteria* (396 species), *Bacteroidetes* (81 species), and the genome of *Candidatus* A. phosphatis. Four training sets were used to bin SLU: the longest contig belonging to *Candidatus* A. phosphatis in the SLU dataset (subsequently removed from the set to be binned), betaproteobacterial isolate genomes, all gammaproteobacterial isolate genomes, and all genomes of *Bacteroidetes*. Scaffolds assigned to each bin were then cross-validated using their existing Blast-based class assignment in IMG/M. As part of the processing pipeline in IMG/M ,the phylogenetic distribution for the metagenome is computed by aligning genes on scaffolds (using BLASTP) to the non-redundant database of sequences computed from isolate genomes stored in IMG. Results are viewable as a phylogenetic distribution of genes in the metagenome by assigning scaffolds to appropriate bins at various taxonomic levels based on the alignment of genes present on them. Results are outlined in Figure 1 Approximately 91% of the scaffolds in the *Candidatus* A. phosphatis bin have best BLAST matches to *Betaproteobacteria*, as do 77% of the scaffolds in the *Betaproteobacteria* bin. Similarly,90% of the scaffolds in the *Bacteroidetes* bin have BLAST matches to *Bacteroidetes*, while the scaffolds in the *Gammaproteobacteria* bin are distributed between *Betaproteobacteria* (59%) and *Gammaproteobacteria* (25%). The latter misclassification could be attributed to the fact that the *Gammaproteobacteria* in the SLU dataset are dominated by *Xanthomonadales* whose scaffolds have high GC content (64-67%) that is closer to that of *Betaproteobacteria* (62%) than to *Gammaproteobacteria* (48%). Moreover the taxonomic position of *Xanthomonadales* is not well defined [9]. This example illustrates the dangers of relying on isolate genome sequences as a training set, especially when relatively large taxonomic groups, such as phyla or classes are considered. Binning can often produce more accurate results if longer contigs from the sequence set to be binned, whose origins are known, are used as training sets.

**Figure 2:**
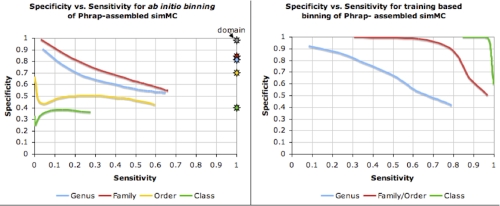
(Left) Sensitivity and specificity on binning contigs longer than 1000 bps in the Phrap-assembled simMC dataset at the genus, family, order, and class levels using ClaMS in an *ab initio* manner. The stars in matching colors indicate the same values for binning all contigs longer than 8,000 bps in the same dataset. The grey star represents the sensitivity/specificity values at the domain level. (Right) Results of binning the same contigs in simMC using user-specfied bins for training.

**Figure 3:**
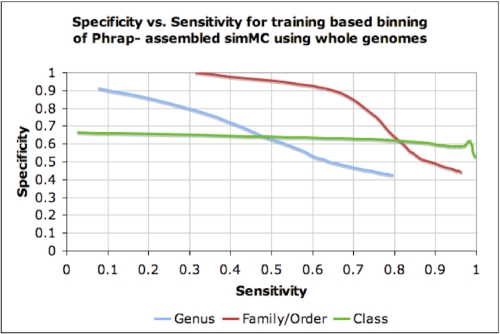
Sensitivity and specificity on binning contigs longer than 1,000 bps in the Phrap-assembled simMC dataset at the genus, family, order, and class levels using ClaMS in an ab initio manner. Complete genomes were used to train the 9 genera, 8 families/orders, and 6 classes specified by the user. Observe that a large amount of noise is added to the bins at the class level because of including all complete genomes in that class. The in-built taxonomy browser in ClaMS was used to make these bin selections.

The Phrap-assembled simulated acid mine drainage dataset (simMC) from FAMeS was binned in an unsupervised manner at various phylogenetic levels. The dataset has been constructed from the reads collected from genomes classified to 79 genera, 60 families, 42 orders, 17 classes, and 9 phyla under the bacterial and archaeal domains. Whole genome sequences of organisms under a taxonomic unit were used to train the bin for that taxonomic unit. For example, all *Alphaproteobacteria* species (except those used in the simulated dataset) were used to train the *Alphaproteobacteria* bin. All contigs longer than 1,000 bases were binned using ClaMS. Figure 2 (Left) illustrates the sensitivity and specificity of the unsupervised binning process at various phylogenetic levels when the best two bins for a contig are considered for the correct match. For example, at the genus level, 79 bins (one for each genus) were used to bin the assembled contigs, where a bin for a particular genus was trained using genomic sequences from all isolate genomes belonging to that genus. Negatives were determined by counting sequences that could not be binned at given cut-offs for distance and contig length. Sensitivity was computed as the percentage of sequences for which bins existed that were binned correctly (ratio of the number of true positives to the sum of the number of true positives and the number of false negatives) while specificity was computed as the ratio of the number of true negatives to the sum of the number of true negatives and the number of false positives. Unsupervised binning of a metagenomic dataset yields relatively accurate results at the genus, family, and domain levels, but the same cannot be said of the order, class, and phylum levels, where the dispersion in the properties of the signature is much greater and the accuracy of binning is much lower. For metagenomic datasets whose dominant constituent populations are known, supervised binning while training on contigs from the same dataset is the best course of action. This is illustrated by the specificity vs. sensitivity plots in Figure 2  (Right), where binning was done on all contigs longer than 1,000 bases in the simMC dataset using training sets specified by the user. A total of 9 genera, 8 families/orders and 6 classes were selected and each bin was trained using contigs from the same metagenome. A combination of the two binning approaches, in which the user specified a training set of isolate genomes instead of selecting training sequences from the same metagenome produces better results than unsupervised binning, but is less accurate than supervised binning with training contigs from the same metagenome (Figure 3).

ClaMS can run in a command-line mode, which makes it convenient to be included in processing pipelines and large-scale batch-processing jobs. Screenshots of the ClaMS user-interface and a demonstration of the usage including visualization of results are available at http://clams.jgi-psf.org. The user-friendly interface, built-in taxonomy browser, bundled genomic signatures, and fast computations make ClaMS an ideal desktop supervised binning application for biologists.

## 

## References

[1] Huson DH Auch AF Qi J Schuster SC MEGAN analysis of metagenomic data. Genome Res 2007; 17:377-386 10.1101/gr.5969107 17255551 PMC1800929

[2] McHardy AC Martin HG Tsirigos A Hugenholtz P Rigoutsos I Accurate phylogenetic classification of variable-length DNA fragments. Nat Methods 2007; 4:63-72 10.1038/nmeth976 17179938

[3] Teeling H Waldmann J Lombardot T Bauer M Glöckner FO A web-service and a stand-alone program for the analysis and comparison of tetranucleotide usage patterns in DNA sequences. BMC Bioinformatics 2004; 5:163 10.1186/1471-2105-5-163 15507136 PMC529438

[4] Heath LS, Pati A. Genomic signatures in de Bruijn chains. WABI 2007, LNBI 4645, 216-227.

[5] Brady A Salzberg SL Phymm and phymmbl: metagenomic phylogenetic classification with interpolated markov models. Nat Methods 2009; 6:673-676 10.1038/nmeth.1358 19648916 PMC2762791

[6] Markowitz VM Ivanova NN Szeto E Palaniappan K Chu K Dalevi D Chen IM Grechkin Y Dubchak I Anderson I IMG/M: a data management and analysis system for metagenomes. Nucleic Acids Res 2007; 36:D534-D538 10.1093/nar/gkm869 17932063 PMC2238950

[7] Mavromatis K Ivanova N Barry K Shapiro H Goltsman E McHardy AC Rigoutsos I Salamov A Korzeniewski F Land M Use of simulated data sets to evaluate the fidelity of metagenomic processing methods. Nat Methods 2007; 4:495-500 10.1038/nmeth1043 17468765

[8] García Martín H Ivanova N Kunin V Warnecke F Barry KW McHardy AC Yeates C He S Salamov AA Szeto E Metagenomic analysis of two enhanced biological phosphorus removal (EBPR) sludge communities. Nat Biotechnol 2006; 24:1263-1269 10.1038/nbt1247 16998472

[9] Comas I Moya A Azad RK Lawrence JG Gonzalez-Candelas F The Evolutionary Origin of Xanthomonadales Genomes and the Nature of the Horizontal Gene Transfer Process. Mol Biol Evol 2006; 23:2049-2057 10.1093/molbev/msl075 16882701

